# Electrophysiological responses of the rice leaffolder, *Cnaphalocrocis medinalis* , to rice plant volatiles

**DOI:** 10.1093/jis/14.1.70

**Published:** 2014-01-01

**Authors:** Xiao Sun, Zhuang Liu, Aijun Zhang, Hai-Bo Dong, Fang-Fang Zeng, Xiang-Yu Pan, Yongmo Wang, Man-Qun Wang

**Affiliations:** 1 Hubei Insect Resources Utilization and Sustainable Pest Management Key Laboratory, College of Plant Science and Technology, Huazhong Agricultural University, Wu han430070, P. R. China; 2 Invasive Insect Biocontrol and Behavior Laboratory, Plant Sciences Institute, USDA-ARS, Beltsville, MD 20705- 2350

**Keywords:** electroantennogram

## Abstract

The rice leaffolder, *Cnaphalocrocis medinalis* Guenée (Lepidoptera: Pyralidae), is one of the most destructive pests of rice. Electrophysiological responses of this species to 38 synthetic volatiles known to be released from rice plants (Poaceae: *Oryza* spp.) were studied using the electroantennogram (EAG) method. Compounds that elicited the strongest EAG responses for each physiological condition were selected for EAG dose-response tests at five concentrations. These compounds included: methyl salicylate, heptanol, linalool, cyclohexanol, and 2-heptanone for one-day-old male moths; heptanol, hexanal, (Z)-2-hexen-1-ol, and nonadecane for one-day- old females; methyl salicylate, heptanol, (E)-2-hexen-1-ol, and (Z)-2-hexen-1-ol for three-day- old males; linalool, heptanol, (E)-2-hexen-1-ol, 2-heptanone, and hexanal for three-day-old females; 2-heptanone, cyclohexanol, linalool, heptanol, and methyl salicylate for five-day-old virgin females; and methyl benzoate, (Z)-2-hexen-1-ol, heptanol, linalool, and hexanal for five- day-old mated females. Female and male *C. medinalis* exhibited broad overlap in their EAG responses, and there was no clear difference between male and female EAG responses to different compounds. Statistical analyses revealed that both volatile compound chemical structure and *C. medinalis* physiological condition (age, sex, and mating condition) had an effect on EAG response.

## Introduction

The rice leaffolder, *Cnaphalocrocis medinalis* Guenée (Lepidoptera: Pyralidae), is a migratory rice pest that is widely distributed in humid tropical and temperate regions of Asia, Oceania, Australia, and Africa ( Khan et al. 1988 ; Kawazu et al. 2001 ). It has recently become widespread throughout the major rice-growing regions of Asia and is one of the most damaging pests of rice (Poaceae: *Oryza* spp.) ( Sogawa and Kiyota 1995 ; Inoue et al. 2004 ). The larvae damage plants by folding the leaves and scraping the green leaf tissues within the fold, causing yield loss by reducing photosynthetic activity ( Nathan et al. 2004 ). Chemical treatments against larvae are often impracticable due to the larvae’s cryptic feeding habit. Effective control strategies must be based on controlling or killing adults.

Despite extensive research on *C. medinalis* response to sex pheromones and implementing related behavioral manipulation techniques ( Ganeswara Rao et al. 1993 ; Ganeswara Rao et al. 1995; Kawazu et al. 2002 ; 2005; 2009), little is known about their hostplant volatile selection *.* Many phytophagous insects use odors as cues for orientation to food resources for their nutrition, mating location, or depositing their offspring ( Jyothi et al. 2002 ; van Der Goes et al. 2006 ; Gallego et al. 2008 ; Fettig et al. 2009 ; Hu et al. 2009 ; Zhuge et al. 2010 ). Ramachandran et al. (1990) examined the electroantennogram (EAG) response of two-day-old *C. medinalis* males and four-day-old *C. medinalis* females to a spectrum of volatile chemicals and identified several compounds that elicit high EAG responses. However, many of the compounds used are not present in rice plants. It has also been shown that the attractiveness of a volatile may be dependent on environmental conditions (e.g., photoperiod or temperature) or the physiological condition of the individual (e.g., age or mating condition), not just the nature of the chemical (reviewed by Anton et al. 2007 ). Therefore, it is necessary to investigate the EAG responses of *C. medinalis* to volatile compounds known to be released from rice plants based on their physiological condition.

Below temperatures of about 25°C, *C. medinalis* females reach sexual maturity three days after emergence and begin mating with males, which reach sexual maturity one day after emergence ( Kawazu and Tatsuki 2002 ). The adults suck nectar or honeydew secreted by aphids as supplementary food and have lifespans of five to eight days. Oviposition usually begins within 24 hrs of mating. *C. medinalis* tends to lay eggs scattered either on front or back of light or dark green rice leaves. Single eggs can be laid by both virgin and mated females, but unfertilized eggs cannot hatch ( Zhang and Wang 1987 ).

This study investigates the EAG responses of *C. medinalis* to volatile compounds known to be released from intact rice plants ( Lou et al. 2005 ; Wechgama et al. 2008 ; Lu et al. 2010 ; Yan et al. 2010 ) based on the age of males and females and the mating condition of females. In addition, dose-dependent EAG response’s relationships to selected compounds were investigated. This research is therefore an important step towards understanding the role of olfaction in manipulating the behavior of *C. medinalis* .

## Materials and Methods

### Insects

*C. medinalis* pupae were collected from an experimental plot in Wu’xue County of Hubei Province in China (115° 33 E; 29° 51 N) in 2010. Sexed pupae were kept inside culture dishes (20 cm diameter) in an environmental chamber (25 ± 1oC, 75 ± 5% RH, 16:8 L:D photoperiod) until the moths emerged. Emerged moths were fed with a 10% sucrose solution in wooden cages (30 x 30 x 30 cm) separate from the larvae. To obtain mated moths, newly emerged male moths and four-day-old female moths were paired in the same cage and allowed to mate. Moths used for EAG studies were of six different physiological conditions: one- and three-day-old male and female moths and five-day-old virgin and mated females.

### Synthetic compounds

Synthetic compounds used in EAG studies were purchased from commercial sources (Fluka, Sigma-Aldrich, ( www.sigmaaldrich.com ; Table 1 ). These compounds belonged to different chemical classes: green leaf volatiles (hexanal, (E)-3- hexen-1-ol, (E)-2-hexenal, (E)-2-hexen-1-ol, (Z)-2-hexen-1-ol ， heptanol, 2-heptanone), alkanes (octane, tetradecane, hexadecane, heptadecane, octadecane, nonadecane, eicosane, henicosane), hydroxyl and carbonyl compounds (cyclohexanol, nerolidol, (E)-2- penten-1-ol, dodecanal), aromatic compounds (p-cymene, benzaldehyde, *N* -phenyl-1- naphthylamine, methyl benzoate, methyl salicylate), and terpenoids (terpinene-4-ol, (1R)- (+)-α-pinene, sabinene, α-terpinene, γ- terpinene, β-myrcene, (-)-(Z)-caryophyllene, (-)-α-cedrene, linalool, lonone, (Z)-farnesene, α-terpineol, cedrol, R-(+)-limonene; Table 1 ). Compounds were stored in accordance with the instructions for use and used within the period of validity.

**Table 1.**
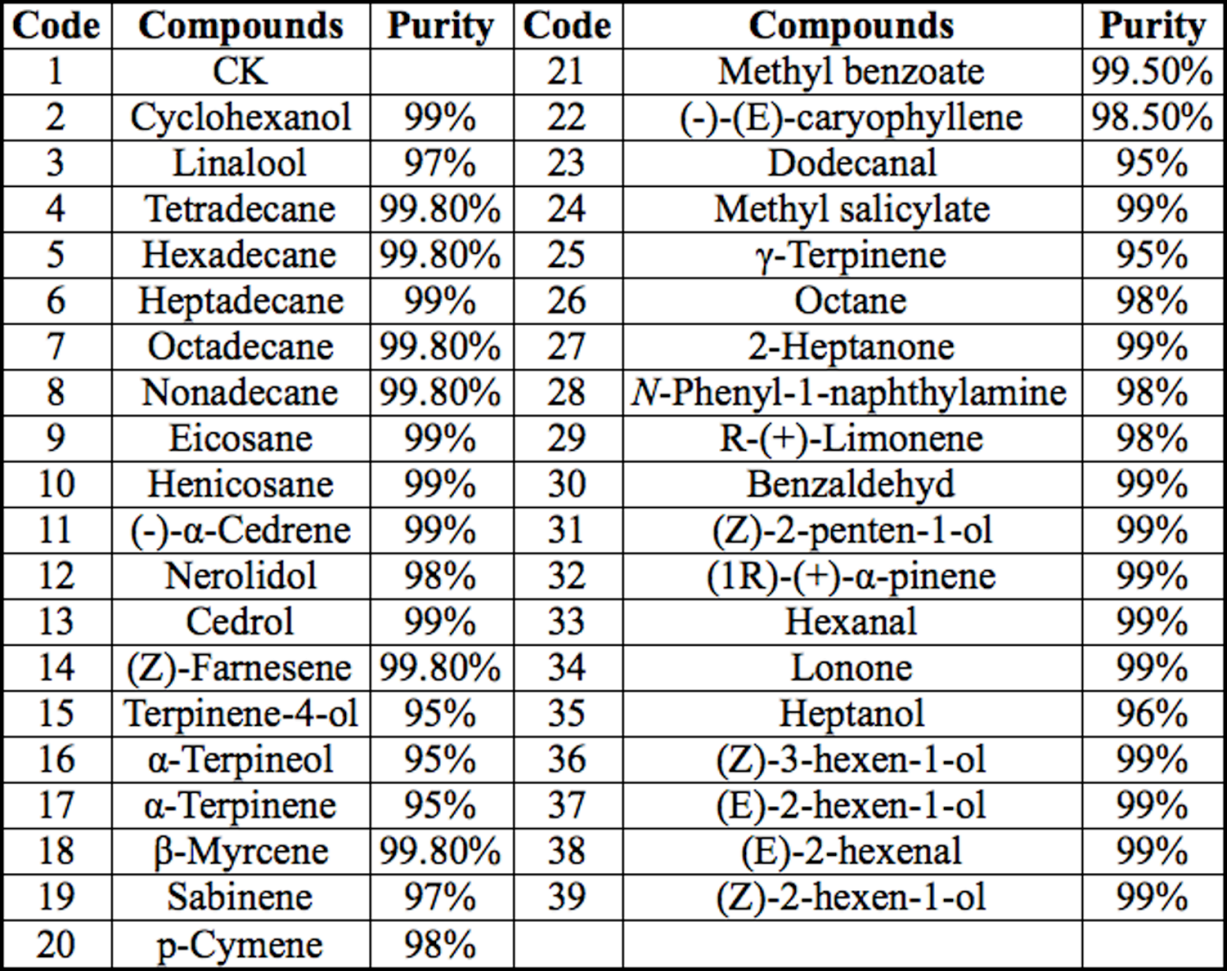
List of compounds tested for *Cnaphalocrocis medinalis* EAG response to rice (Poaceae: *Oryza* spp.) volatile compounds.

### Electroantennogram recordings

The receptivity of antennae from male and female moths to plant volatile compounds was determined by EAG. EAG measurements were made using a commercially available electroantennographic system (Model CS-05; Syntech, ( www.syntech.nl ) consisting of a dual electrode MTP-4 probe for antenna fixation, a CS-05 stimulus controller, and an IDAC box for data acquisition. Antennae were carefully removed at the base, and several terminal segments at the distal end were excised before attaching them to electrodes with Spectra 360 conductive gel (Parker, ( www.parkerlabs.com ). A continuous stream of moistened air was then blown at the antennae through a glass tube (0.8 cm diameter) positioned 1 cm away. Solutions of synthetic compounds were diluted in distilled paraffin oil. Test compounds (10 µL, 10 µg/µL) were applied to a piece of filter paper (5 mm × 20 mm), which was inserted into a Pasteur pipette. The tip of the pipette was inserted about 3 mm into a small hole in the wall of a glass tube (8 mm diameter, 12 cm long) directed at the antennal preparation. An air stimulus controller was used for air and odor delivery. A constant flow of 2 L/min of active carbon-filtered air was passed over the antennae through the open end of the glass tube. 200 mL/min of air was applied through the Pasteur pipette into the main airflow for 0.5 sec, creating an odor stimulation of the antenna. The response to the reference standard, (Z)-3-hexen-1-ol, was measured at the beginning and end of each recording session to correct for the loss of sensitivity of the preparation. It was assumed that the decrease in sensitivity was linear with time for the correction. Data were then normalized to the standard as follows ( Gouinguene et al. 2005 ):

$$rEAG = \frac{EAG(A)}{EAG(std1)+\frac{EAG(std 2)-EAG(std 1)}{RT(std 2)-RT(std1)}\times (RT(A)-RT(std1))}$$

Where rEAG means relative EAG response; EAG(A) is the amplitude (mV) of the EAG response to compound A; EAG(std1) is the EAG response to the standard at the beginning of the recording; EAG(std2) is the EAG response to the standard at the end of the recording; T(A) is the time elapsed before stimulation with compound A; T(std1) is the time of the first stimulation; T(std2) denotes the time of the final stimulation.

### Comparison of EAG responses to different compounds

The compounds included in this study ( Table 1 ) were tested individually as olfactory stimuli. Antennae were stimulated twice with each substance at 30 sec intervals. Fifteen replicates were carried out for each sex, and the sequence of exposure of each antenna to stimulus was randomly defined.

### Dose-response relationship

Four or five compounds that elicited the strongest EAG responses for each sex were selected for EAG dose-response tests. Five doses (0.01, 0.1, 1, 10, and 50 µg/µL) dissolved in liquid paraffin were tested for their electrophysiological activity. Stimulations were made in order of concentration, with the lowest dose first and with 30 sec intervals between stimuli. The liquid paraffin was used as a blank control, and 10 µg/µL of (Z)-3-hexen- 1-ol was used as a reference standard. Ten replicates were carried out for each sex.

### Data analyses

Data were analyzed statistically using SPSS 11.0 for Windows (IBM, ( www.ibm.com ). The relationship of male and female EAG responses was examined by Tukey’s multiple comparison analysis. Paired-sample *t* -tests were used to analyze the EAG differences between males and females or virgin females and mated females.

## Results

### EAG activity of synthetic compounds

**Figure 1.**
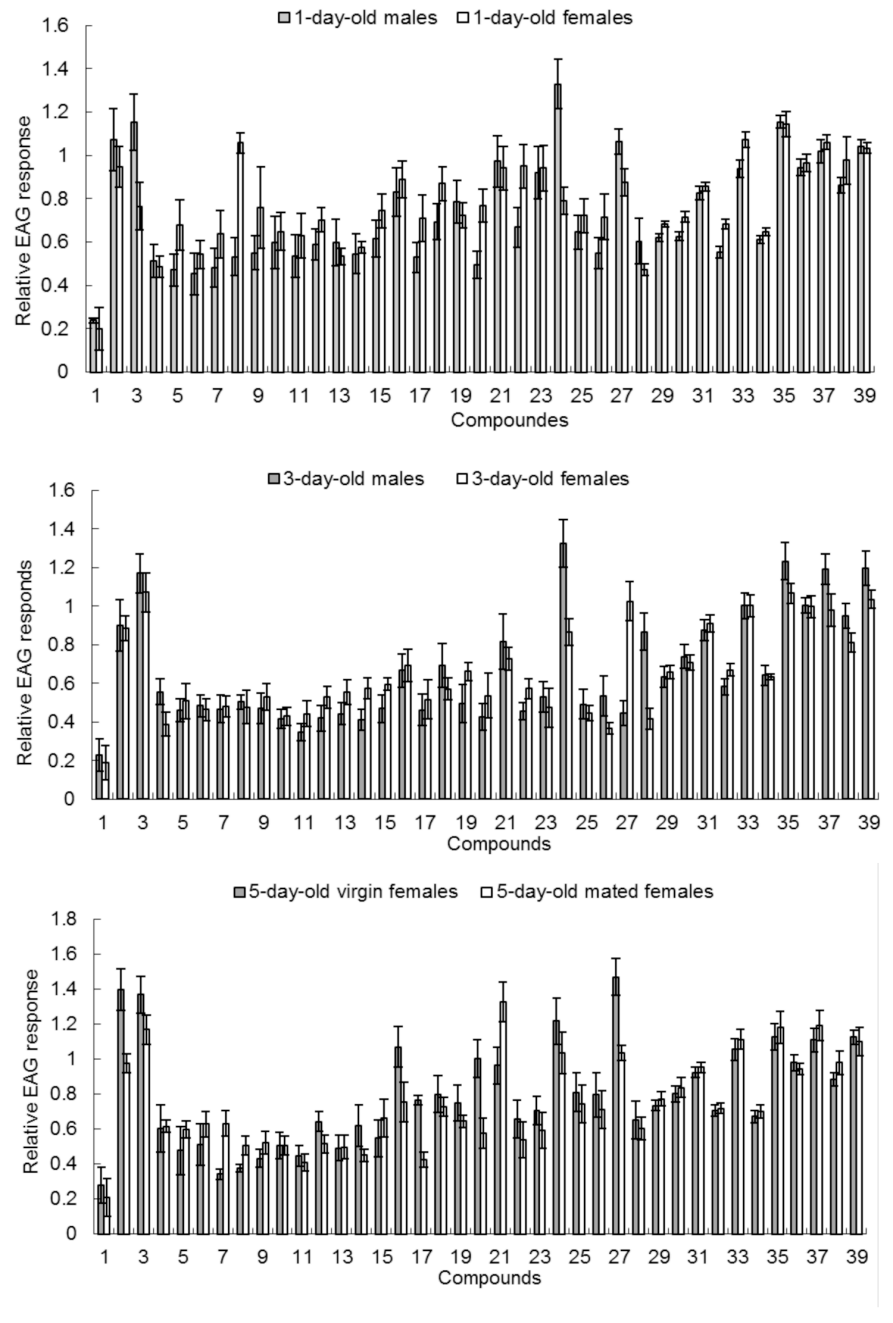
Relative EAG responses (mean ± SE, n = 15) of *Cnaphalocrocis medinalis* to different compounds. Compounds numbers are: 1. CK; 2. cyclohexanol; 3. linalool; 4. tetradecane; 5. hexadecane; 6. heptadecane; 7. octadecane; 8. nonadecane; 9. eicosane; 10. henicosane; 11. (-)-α-cedrene; 12. nerolidol; 13. cedrol; 14. (Z)-farnesene; 15. ter- pinene-4-ol; 16. α-terpineol; 17. α-terpinene; 18. β-myrcene; 19. sabinene; 20. p-cymene; 21. methyl benzoate; 22. (-)-trans- caryophyllene; 23. dodecanal; 24. methyl salicylate; 25. γ-terpinene; 26. octane; 27. 2-heptanone; 28. *N* -phenyl-1-naphthylamine; 29. R-(+)- limonene; 30. benzaldehyde; 31. cis-2-penten-1-ol; 32. (1R)-(+)-a- pinene; 33. hexanal; 34. lonone; 35. heptanol; 36. (Z)-3-hexen-1-ol; 37. (E)-2-hexen-1-ol; 38. (E)-2-hexenal; 39. (Z)-2-hexen-1-ol. High quality figures are available online.

Statistical analyses (ANOVA) revealed that both the chemical structure of volatile compounds and the physiological condition of *C. medinalis* influenced EAG responses. For the six different physiological conditions of *C. medinalis* investigated, all rice plant compounds elicited significantly greater EAG responses than the solvent controls at doses of 10 µg/µL ( *P* < 0.01; Figure 1 ). There were no significant differences in response to stimulation with the reference standard between one- day-old males and females (t = 0.93, *P* > 0.05), between three-day-old males and females (t = 1.07, *P* > 0.05), or between five- day-old virgin and mated females (t = 1.38, *P* > 0.05). However, there was a significantly different EAG response to some compounds between males and females.

For one-day-old moths, the highest EAG responses of virgin males were elicited by methyl salicylate, heptanol, linalool, cyclohexanol, and 2-heptanone. The highest EAG responses of virgin females were elicited by heptanol, hexanal, (Z)-2-hexen-1-ol, and nonadecane. For the three-day-old moths, the strongest EAG responses were elicited by methyl salicylate, heptanol, (E)-2-hexen-1-ol, and (Z)-2-hexen-1-ol for males, and linalool, heptanol, (E)-2-hexen-1-ol, 2-heptanone, and hexanal for females. The highest EAG responses of five-day-old virgin females were elicited by 2-heptanone, cyclohexanol, linalool, heptanol, and methyl salicylate, while the five-day-old mated females exhibited significantly larger EAG responses to hexanal, (Z)- 2-hexen-1-ol, heptanol, linalool, and methyl benzoate ( Figure 1 ).

All green-leaf volatiles elicited stronger antennal responses than most of the alkanes, terpenoids, aromatic compounds, and hydroxyl and carbonyl compounds, and did not elicit significantly different EAG responses between all the physiological conditions of *C. medinalis* .

Statistical analyses revealed that one-day-old *C. medinalis* males exhibited significantly greater EAG responses to nerolidol (t = 3.62, *P* < 0.01), dodecanal (t = 3.35, *P* < 0.01), cedrol (t = 3.35, *P* < 0.01), henicosane (t = 2.70, *P* < 0.05), cyclohexanol (t = 2.70, *P* < 0.05), *N* -phenyl-1-naphthylamine (t = 2.70, *P* < 0.05), (-)-α-cedrene (t = 2.78, *P* < 0.05), sabinene (t = 2.63, *P* < 0.05), and γ-terpinene (t = 2.26, *P* < 0.05) than three-day-old males. Analogously, one-day-old females showed significantly greater EAG responses to hexadecane (t = 2.98, *P* < 0.05), octadecane (t = 2.70, *P* < 0.05), and nonadecane (t = 2.70, *P* < 0.05) than five-day-old virgin females. One- day-old males showed significantly greater EAG responses to the linalool (t = 3.62, *P* < 0.01), 2-heptanone (t = 2.32, *P* < 0.05), and methyl salicylate (t = 2.64, *P* < 0.05) than one-day-old females. In contrast, one-day-old females showed significantly greater EAG responses to (1R)-(+)-α-pinene (t = 3.80, *P* < 0.01), p-cymene (t = 4.07, *P* < 0.01), ter- pinene-4-ol (t = 3.10, *P* < 0.01), hexadecane (t = 2.41, *P* < 0.05), nonadecane (t = 2.19, *P* < 0.05), benzaldehyd (t = 2.65, *P* < 0.05), α- terpinene (t = 2.17, *P* < 0.05), and β-myrcene (t = 2.30, *P* < 0.05) than one-day-old males. In addition, the data also showed that five-day- old mated females exhibited significantly greater EAG responses to hexadecane (t = 3.83, *P* < 0.01), nonadecane (t = 3.73, *P* < 0.01), octadecane (t = 3.28, *P* < 0.05), and heptadecane (t = 2.24, *P* < 0.05), which all belonged to the alkane group, than five-day- old virgin females.

### Electroantennography dose-dependent responses

**Figure 2.**
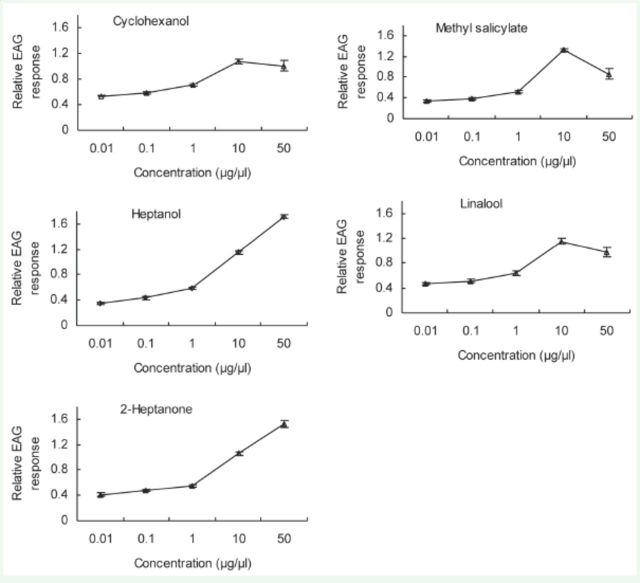
EAG concentration-response (mean ± SE, n = 10) curves of *Cnaphalocrocis medinalis* antennae to methyl salicylate, heptanol, linalool, cyclohexanol, and 2-heptanone for one-day-old males. High quality figures are available online.

**Figure 3.**
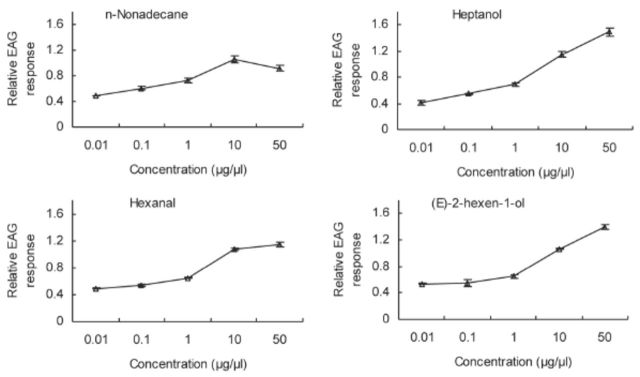
EAG concentration-response (mean ± SE, n = 10) curves of *Cnaphalocrocis medinalis* antennae to heptanol, hexane, (Z)-2-hexen-1- ol, and nonadecane for one-day-old females. High quality figures are available online.

**Figure 4.**
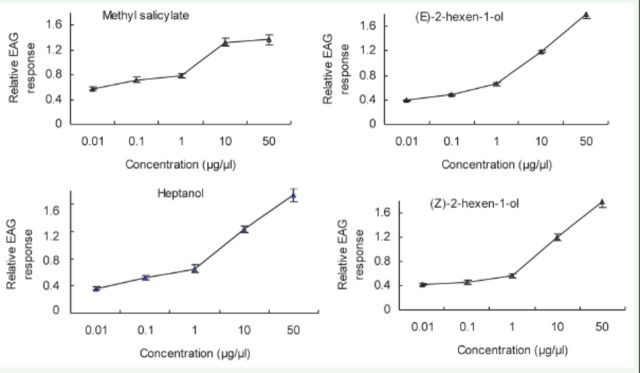
EAG concentration-response (mean ± SE, n = 10) curves of *Cnaphalocrocis medinalis* antennae to methyl salicylate, heptanol, (E)-2- hexen-1-ol, and (Z)-2-hexen-1-ol for three-day-old males. High quality figures are available online.

**Figure 5.**
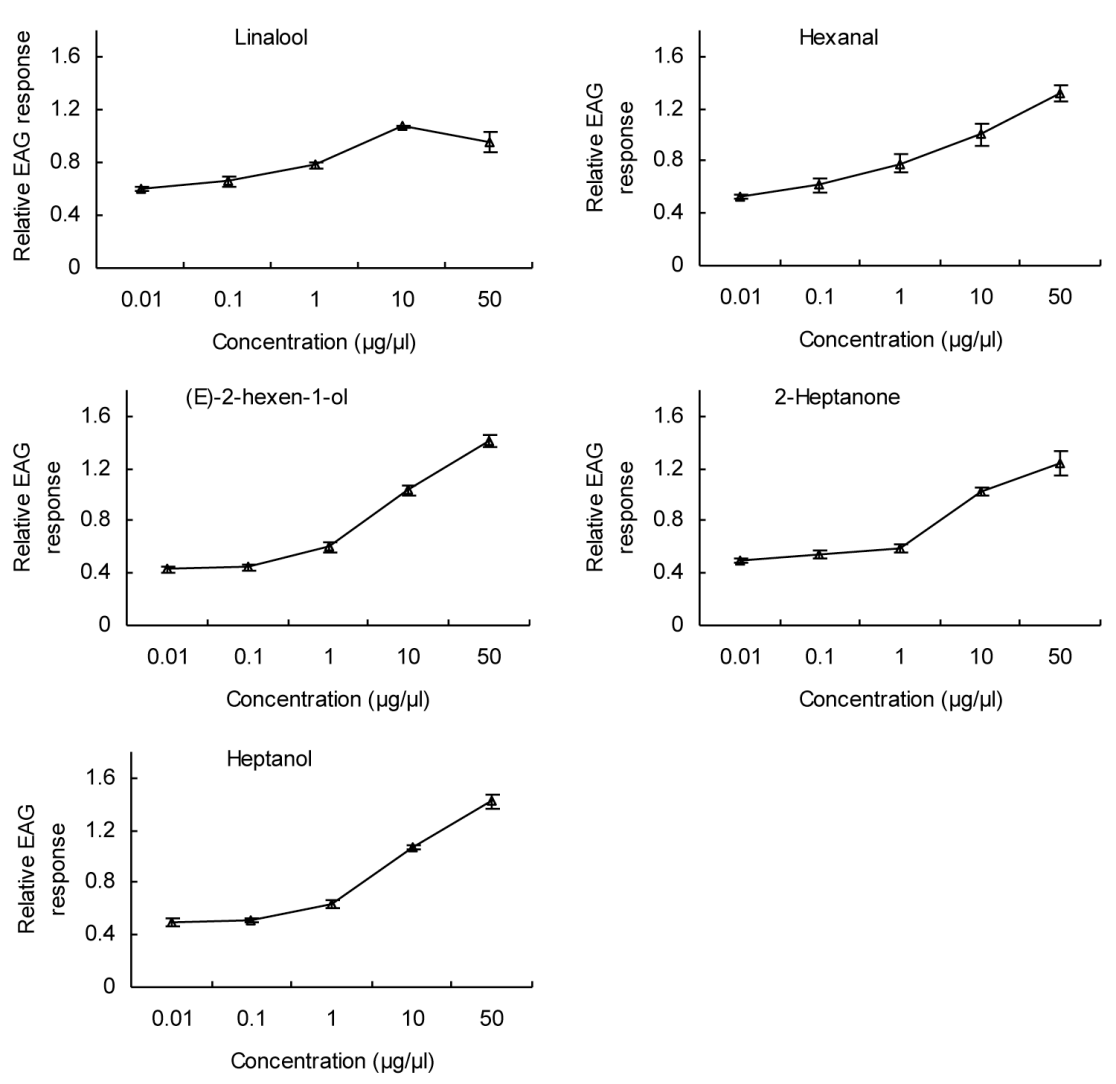
EAG concentration-response (mean ± SE, n = 10) curves of *Cnaphalocrocis medinalis* antennae to linalool, heptanol, (E)-2-hexen-1- ol, 2-heptanone, and hexanal for three-day-old females. High quality figures are available online.

**Figure 6.**
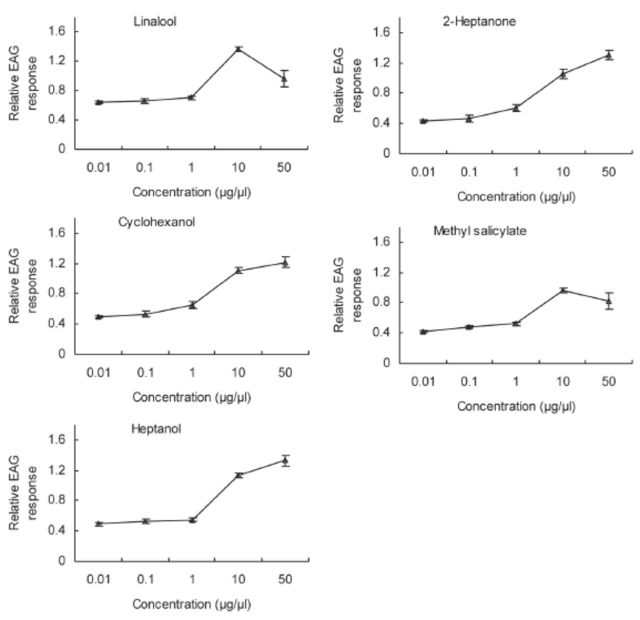
EAG concentration-response (mean ± SE, n = 10) curves of *Cnaphalocrocis medinalis* antennae to 2-heptanone, cyclohexanol, linalool, heptanol, and methyl salicylate for five-day-old virgin females. High quality figures are available online.

**Figure 7.**
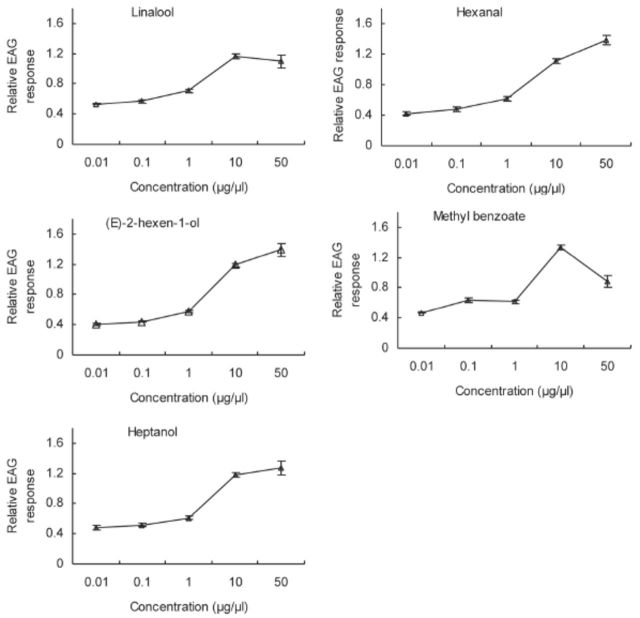
EAG concentration-response (mean ± SE, n = 10) curves of *Cnaphalocrocis medinalis* antennae to methyl benzoate, (Z)-2-hexen-1- ol, heptanol, linalool, and hexanal for five-day-old mated females. High quality figures are available online.

Based on the EAG results, compounds were selected that elicited the strongest responses in each of the six different *C. medinalis* physiological conditions tested in the EAG dose- response tests. The amplitude of the EAG response increased with dose for all compounds until the concentration of the compound was increased from 10 to 50 µg/µL. Between 10 to 50 µg/µL, the amplitude of the EAG response decreased, indicating saturation of receptors at 10 µg/µL ( Figures 2–7 ). These compounds were methyl salicylate, linalool, and cyclohexanol for one-day-old males; *N* -nonadecane for one-day-old females; methyl salicylate for three-day-old males; linalool for three-day-old females; linalool and methyl salicylate for five-day-old virgin females; and linalool and methyl benzoate for five-day-old mated females.

In most cases, no difference was observed in the six physiological groups’ EAG responses to a given compound. However, at a dose of 50 µg/µL, three-day-old males were more sensitive to heptanol and (E)-2-hexen-1-ol than three-day-old females.

## Discussion

Plant volatiles (green leaf volatiles, monoterpenes, and other groups) were chosen based on previous studies demonstrating that they are emitted from undamaged and insect- infested plants ( Takabayashi et al. 1994 ; Loughrin et al. 1995 ; Röse et al. 1996 ; Paré ; and Tumlinson 1998; Rodriguez-Saona et al. 2002 ; Blackmer et al. 2004 ; Williams et al. 2005 ). *C. medinalis* antennae of both sexes and various physiological conditions respond to a wide range of volatiles belonging to different chemical classes: green leaf volatiles, alkanes, terpenoids, aromatic compounds, hydroxyl, and carbonyl.

Compounds that elicited strong EAG responses included (Z)-3-hexen-1-ol, (E)-2-hexen-1- ol, (E)-2-hexena, (Z)-2-hexen-1-ol, heptanol, hexanal, cyclohexanol, linalool, cyclohexanol, nonadecane, and methyl salicylate. These compounds could play an important role in hostplant location. Additionally, these compounds have previously been reported to elicit high EAG responses ( Ramachandran et al. 1990 ), except for (Z)-3-hexen-1-ol, hexanal, (Z)-2-hexen-1-ol and (E)-2-hexena. Cyclohexanol and nonadecane were not previously tested. Some compounds that have been reported to elicit high EAG responses, such as terpinene-4-ol and α-terpineol ( Ramachandran et al. 1990 ), did not elicit high EAG response in *C. medinalis* in our experiment.

Female and male *C. medinalis* exhibited broad overlap in their EAG responses to individual plant odors, and there was no clear pattern of difference between responses of female and male antennae to different compounds. For example, strong EAG responses were elicited by heptanol in *C. medinalis* moths of all six physiological conditions. Likewise, (Z)-2- hexen-1-ol elicited strong EAG responses in all of the females, while all of the tested males exhibited sensitivity to methyl salicylate. Females showed significantly greater response than males to 2-heptanone and nonadecane, while males showed significantly greater response than females to linalool and methyl salicylate. In most cases, there were also differences between the male and female EAG responses to some plant odors at higher doses. Different EAG responses between females and males might be indicative of sex-specific differences in the importance of certain volatile cues in hostfinding ( Light et al. 1988 ). It has been reported that for many phytophagous insects, even though females and males can detect the same range of volatiles, there are differences between the EAG responses of the two sexes ( Hansson et al. 1989 ; Ramachandran et al. 1990; Chinta et al. 1994 ; Raguso et al. 1996 ; Groot et al. 1999 ; Zhang et al. 2000 ; Fraser et al. 2003 ). Indeed, while both (Z)-3- hexenol (Z3-6:OH) and manuka oil emitted by ash foliage attracted *Agrilus planipennis,* the former was more attractive to males and the latter had a greater effect on females ( Grant et al. 2010 ). For *C. medinalis* , different EAG responses between sexes may be related to possible differences in the antennal morphology of the sexes. There are morphological differences between the type, size, and number of antennal sensilla in *C. medinalis* females and males. The antennae of males are longer and larger than those of females, there are more sensilla basiconc and sensilla coeloconica in males than females, and sensilla styloconica II are longer and thinner in males and exhibit surface longitudinal grooves, in contrast to their smooth surface in females ( Sun et al. 2011 ). In contrast, Mercader et al. (2008) reported no significant sexual differences in the EAG responses of *Papilio glaucus* and *P. canadensis* to hostplant odor.

Differences in EAG responses were noted with respect to the age of the moths. One-day- old males showed significantly greater EAG responses than three-day-old males to dodecanal and cyclohexanol. One-day-old females showed significantly greater EAG responses to nonadecane than five-day-old virgin females. There are very few reports about age- dependent changes in olfactory-guided behavior to host plants. However, some male moths have been shown to change their responsiveness to sex pheromones with age. When testing the behavioral responsiveness of *Agrotis ipsilon* males to sex pheromone, clear changes were found with age; freshly hatched males did not react to the conspecific female’s pheromone, but after a few days some males did respond and the best responses were obtained within about five days ( Gadenne et al. 1993 ). The response to the male-produced aggregation pheromones changed with adult age in *Schistocerca gregaria* , and adults were indifferent to aggregation pheromones from three to four weeks of age ( Ignell et al. 2001 ). Newly emerged *C. medinalis* were likely more sensitive to plant volatiles due to higher nutritive needs. This could also make it easier for one-day-old males to find mates. Some plant volatiles are perceived in a synergistic manner and employed by insects to locate mates and increase reproduction ( Reddy and Guerrero 2004 ). Interactions between pheromones and plant volatiles have also been observed in the laboratory and field ( Dickens et al. 1993 ; Light et al. 1993 ; Reddy and Guerrero 2000 ).

Mating condition also affected female EAG response. The data show that five-day-old mated females exhibited significantly greater EAG responses than five-day-old virgin females to hexadecane, nonadecane, octadecane, and heptadecane, which all belonged to the alkane group.The greater response of mated females may be adaptive, given that these compounds are associated with the presence of host eggs. Many insects undergo significant changes in their general physiology during mating; this plasticity in host recognition and acceptance is suggested to be very important in insect speciation ( Berlocher and Feder 2002 ; Linn et al. 2003 ; Tasin et al. 2007 ). Mated herbivorous females were thought to be more likely to search for their host plant in order to lay their eggs ( Anton et al. 2007 ). It has been reported that only mated *Lobesia botrana* females were attracted by grapevine plant volatiles ( Masante-Roca et al. 2007 ).

The amplitude of some volatiles decreased when the concentration of the compound was increased from 10 to 50 µg/µL, indicating saturation. The EAG responses to lower doses of the extract may well correspond to attraction, whereas the EAG responses to higher doses may correspond to repellency or behavioral inhibition. This proposal is consistent with the view that the mechanisms of hostplant selection in insects are largely a matter of gradation and balance between chemicals rather than clearly definable and different cues (Schoonhoven et al. 2005). This idea suggests that *C.medinalis* may rely on various EAG-active compounds in particular ratios to find the plant for feeding or for oviposition. Bruce et al. (2005) argued that the ratio between ubiq- uitous plant volatiles should be seen as the most prevalent mechanism mediating hostplant recognition. Many studies demonstrate that host location can be identified by the particular blend of electrophysiologically active volatiles identified from the headspace of an insect’s host plant ( Guerin et al. 1983 ; Nojima et al. 2003 ; Hern and Dorn 2004 ; Tasin et al. 2007 ; Tasin et al. 2010 ). Also, an EAG response does not necessarily mean there is a behavioral response. Behavioral bioassays showed that not all higher EAG-active compounds, when tested individually, affected behavioral activity of *Batocera horsfieldi* (M- Q Wang, personal observation). EAG-active compounds could be attractive or repellent to an insect. It has been demonstrated that the green leaf volatile Z-3-hexen-1-ol, which elicited strong EAG responses in *Arhopalus tristis* , was a repellent that reduced attraction and oviposition ( Suckling et al. 2001 ). Based on this electrophysiological data, *C. medinalis* EAG and behavioral responses to the tested mixtures of compounds should be studied further.

## Abbreviations

EAG: electroantennogram
